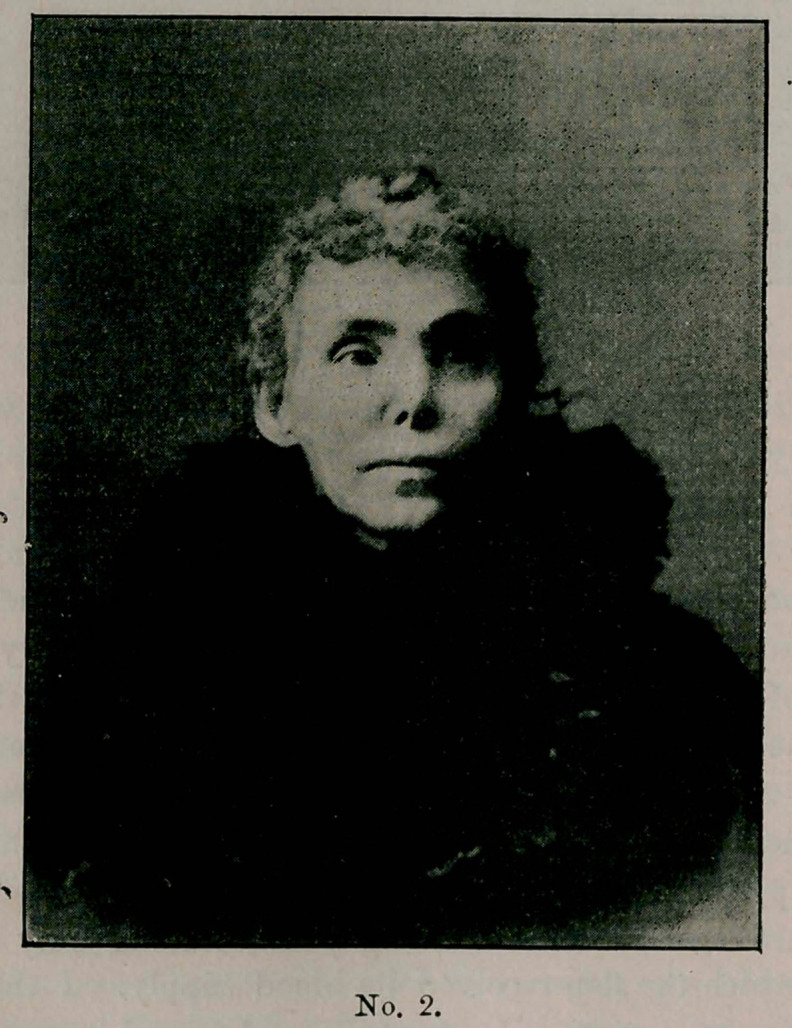# Report of a Successful Rhinoplastic Operation for Loss of the Nose

**Published:** 1897-08

**Authors:** J. M. Crawford

**Affiliations:** Atlanta, Ga.


### REPORT OF A SUCCESSFUL RHINOPLASTIC OPERATION FOR LOSS OF THE NOSE. ALSO OPERATION FOR REMOVAL OF THE DRUM-MEMBRANE AND NECROSED BONES IN CHRONIC SUPPURATIVE AURAL CATARRH.

BY J. M. CRAWFORD, M.D.,

ATLANTA, GA.

The patient, a maiden lady of forty years of age, first came under my notice about ten years ago. She was then suffering from a severe case of syphilitic iritis, which responded promptly to my treatment, her vision being restored. The treatment was continued more or less regularly for a year or so.

Some three years ago, however, there developed a catarrhal condition of the nose. Her general health having failed, and being a woman of moderate means, she was compelled to remove to the country, which placed her beyond the reach of medical attention. She did not suspect the catarrhal trouble had any connection with her former ailment until after the whole of the septum, cartilage, integument, and turbinated bones had all sloughed away, leaving only the tip of the nose intact, as shown in sketch No. 1.

At the time she presented herself the opening was oval in shape, and extending downward and to the left side about an inch and a half long. She was indeed in a pitiable condition, having this enormous and unsightly aperture in her face, which was a source of great annoyance, as the air would strike against the mucous membranes, keeping up a constant irritation, and drying the secretions of the nasal cavity, hardening them into coatings or crusts that she could not remove, such enormous quantities collecting in a surprisingly short time. The condition of the case was rendered more serious and treatment more imperative since the hearing was being greatly impaired, caused by the air striking the pharyngeal mouths of the Eustachian tube through this opening, which was such as to give

*Read before Georgia Medical Association, April, 1897.

rise to a reasonable fear that she might become permanently deaf. Under these conditions an operation to close the opening was determined upon.

No. 1.

My first effort was to secure healing of the ulceration, which was effected by the use of sprays and other cleansing processes, together with large doses of the iodides.

On November 1, 1896, assisted by Drs. W. S. Goldsmith, E. L. Brooks and Prof. Shaefer, the patient being under the influence of ether, I pared off the margins of the cavity, avoiding any unnecessary loss of tissue. Then with a sharp bistoury, commencing at the apex of the opening, I made an upward and oblique incision over the forehead, reaching nearly to the hair, then turning across more or less parallel to the margins of the hair for two inches from the base of the flap; from this point the incision was continued downward and inward nearly to the starting point, leaving only a pedicle through which the flap received its blood supply. I then peeled off this triangular piece of integument, and turned it over the aperture, adjusting its margin to the denuded surface around the opening, in a manner reproducing the original outlines of this portion of the nose. The cut edges of the integument upon the forehead were drawn as close together as possible, leaving a denuded space the greatest width of which was two inches in breadth. It was dressed with iodoform gauze; the nasal cavity was also packed with the same material. The dressing was renewed daily for two weeks. She was then again placed under the influence of ether for the purpose of clipping the pedicle and relieving it of the twist in which it had been placed. And putting this disengaged flap upon a stretch I carried it as far as possible over the denuded portion of the forehead and then fastened it there and then dressed it as before. In a reasonable time the entire healing was completed, the patient presenting herself as you see her in sketch No. 2.

No. 2.

When first considering the operation, a question arose as to where the necessary quantity of integument should be taken. The size of the opening would render it impossible to get a graft from the arm or other parts of the body that would live. This determined me to take the flap from the forehead. The integument removed from the forehead has been entirely replaced, leaving only a small cicatrix which would be scarcely observed except on close scrutiny.

The operation was eminently successful. The points of interest briefly stated are as follows:

1. Since the operation the patient is able, by the use of a little salt water drawn from the hand into the nose, to keep the nasal cavity clean.

2. The patient's hearing has been greatly improved.

3. The catarrhal symptoms are much better and less annoying.

4. The removal of an unsightly facial deformity has decidedly improved her personal appearance and rendered her company less objectionable.

*Case 2.*—Mr. T., aged twenty-six, presented himself for treatment for chronic purulent otitis media, about the first of June, 1896, and gave the following history: Six years before, while in Chicago at college, during a very severe winter, he took a violent cold which resulted in acute suppurative aural catarrh in left ear. This was followed by mastoiditis, for which he was operated on at that time. The discharge was kept up all the while, since resulting in an entire loss of hearing in this ear.

Since he had tried antiseptic applications thoroughly during this time and finding no material benefit, I lost but little time telling him of the operation I desired to make, the removal of the drum-membrane, together with the necrosed bones. This I did the first of July, 1896, he, of course, being under ether.

To secure the necessary illumination of the inner ear I adopted the following plan: The patient was placed prostrate in an ordinary Wilkerson dental chair, and having on my forehead a head mirror I arranged my electric light so that sufficient light could be thrown into the ear canal. I first punctured the drum-membrane near the lower portion of the posterior wall of the canal with a sharp-pointed bistoury. Then introducing a probe-pointed knife, I made a circular incision around the margin of the membrana tympani, severing it from the ear canal. With the forceps I then removed the membrane and malleus. I found the malleus in a healthy condition. Then with a curette I dislodged two small pieces of necrosed bones, probably the incus and stapes. It was then made evident that these necrosed bones were keeping up the formation of pus. The necessity for the operation was made more evident, since the remaining portion of the perforated membrane had served as a shield or blind behind which pus had constantly accumulated and made difficult the necessary drainage.

The operation then secured a twofold advantage; first, the removal of the cause; second, a perfect drainage which was necessary for the healing to take place.

After the operation followed the usual antiseptic treatment. The patient was put to bed and there remained for two or three days.

The discharge from the ear continued for several weeks, constantly growing less until it finally ceased, and now for several months has been entirely well.

The operation was not intended to benefit the hearing, but to get rid of a constant discharge from the ear, which was not only annoying to the patient and required great care in washing and cleansing, but was also a constant menace to his health, rendering a probable return of the mastoiditis from which he had previously suffered.

The operation has proved a success. The patient is now entirely well, and the discharge from the ear, which has continued constantly for more than six years, has ceased altogether.

---

The long, gloomy operating-room of the hospital is hushed and still; soft-voiced, gentle-eyed nurses move quickly here and there, and a skillful attendant arranges the cruel-looking instruments upon a table. Before administering chloroform to the patient, prior to the amputation, the kindly doctor leans over and asks him if he has any message for his friends. "Naw!" he murmurs wearily; "jest tell 'em dat you saw me, an' dat I'm losin' flesh."—*Sun.*

## Figures and Tables

**No. 1. f1:**
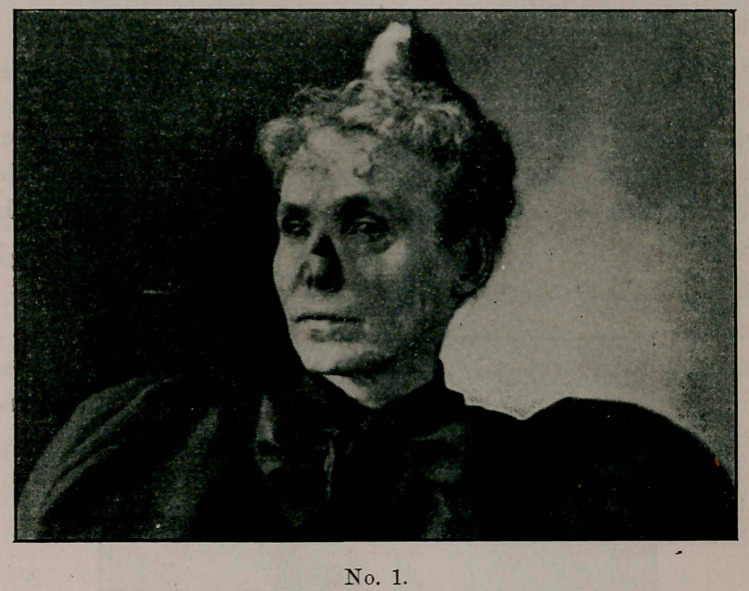


**No. 2. f2:**